# Giant Lipoma of Posterior Neck with Bleeding Decubitus Ulcer: A Rare Entity

**DOI:** 10.4103/0974-2077.69027

**Published:** 2010

**Authors:** Satyajeet Verma, Manish Varma, Sanjay Kala, RK Singh

**Affiliations:** *Department of Surgery, G.S.V.M. Medical College, Kanpur, Uttar Pradesh - 208 002, India*

**Keywords:** Giant lipoma, pressure ulcer, bleeding

## Abstract

Giant lipomas are benign soft tissue tumours. They are found relatively rarely on the posterior part of the neck. Bleeding pressure ulcer in this giant tumour is a rare presentation. Surgical interventions in these tumours are very challenging because, sometimes, extension to the spinal cord and malignant change may occur, especially in old age. Knowledge of the anatomy and meticulous surgical techniques are needed for such giant lipomas.

## INTRODUCTION

Lipomas infrequently occur in the head and neck. Giant lipomas are defined by Sanchez *et al*. as lesions with size of at least 10 cm in one dimension or weighing a minimum of 1,000 g.[1] A large neck mass (>10 cm) with a rapid growth rate should raise concerns about a possible malignancy.[1] The presentation with pressure sore is uncommon with a giant lipoma. Surgical excision of a lipoma is often used as the definitive treatment modality. In the present report, a 60-year-old man who presented with a giant neck lipoma with episodic bleeding and pressure ulcer is described. The 22 cm × 12 cm mass was successfully removed. The surgery produced excellent cosmetic results and no functional impairment.

## CASE REPORT

A 68-year-old male presented to our surgical outpatient department with a huge lump at the back of his neck for the last 11 years. There was an ulcer at the top of the lump for the last 9 months. Episodic bleeding was also reported from the ulcer for the last 6 months.

**Figure 1:**
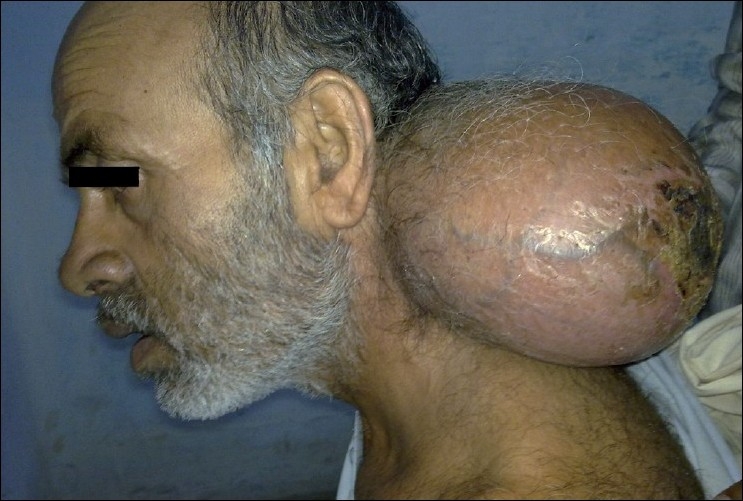
Photograph of the patient showing giant lipoma at the posterior triangle

On local examination of the neck, there was a 22 cm × 12 cm-sized swelling at the posterior side of the neck [Figure 1]. It was nontender and soft to firm in consistency. There was a 6 cm × 5.5 cm decubitus ulcer at the top of the lump. Dilated veins were present in the skin around the decubitus ulcer. There was no regional lymphadenopathy.

Fine needle aspiration revealed mature lipocytes indicative of lipomatous lesion. Contrast Enhanced Computed Tomography Scan of the neck revealed a giant subcutaneous lipoma at the posterior triangle without septations. There was no communication with the spinal cord.

**Figure 2:**
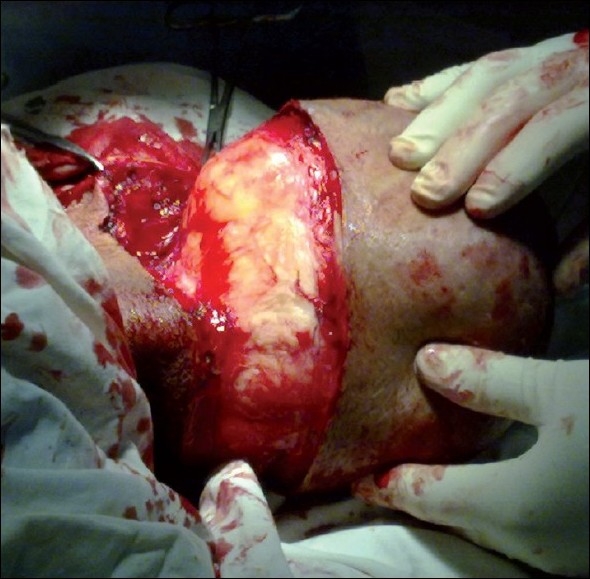
Operative photograph showing lump excision with raised upper and lower skin flaps

**Figure 3:**
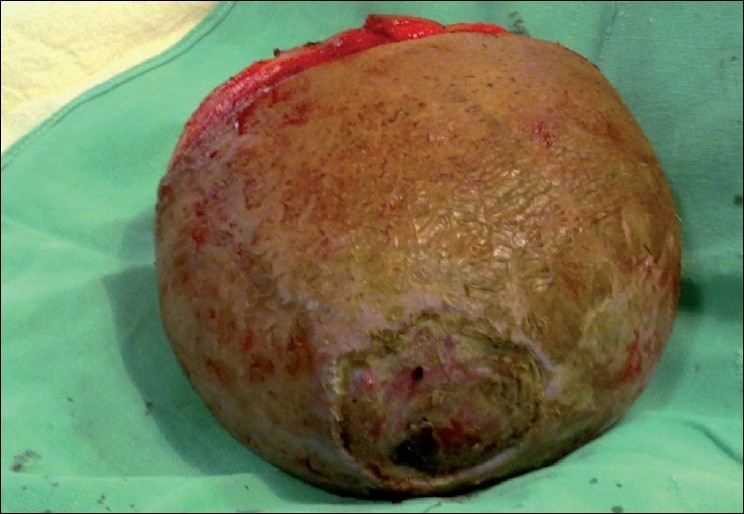
Excised lipoma with decubitus ulcer

After intubation with general anaesthesia, the patient was positioned in a prone position. An elliptical transverse incision of 12 cm was given at around the base of the lump. The superior and inferior skin flaps were raised [Figure 2]. Separation of lipoma from the surrounding tissues was easy and was performed with sharp and blunt dissections. Five to six large feeding vessels were also ligated to isolate the lipoma. The redundant skin was removed and the upper and lower skin flaps were stitched together with Silk-3-0 after securing haemostasis and placing a suction drain. The resected mass was 2.2 kg in weight and 22 cm × 12 cm in diameter [Figure 3]. The postoperative period was uneventful. The drain was removed after 3 days and the patient was discharged on the 9^th^ postoperative day.

Histopathological analysis of the resected mass revealed mature, proliferative lipocytes with no cellular atypia, and it was diagnosed as benign giant lipoma.

## DISCUSSION

Lipomas are the most common mesenchymal tumours.[2] Only approximately 25% of the lipomas and their variants arise in the head and neck region.[3] Common locations for lipomas are the back, arm, shoulder, anterior chest wall, breast, thigh, abdominal wall, legs, forehead and face, in decreasing order of frequency.[4] Of those lipomas that occur in the head and neck region, the most common location is the posterior neck. Those tumours are more common in women and occur usually in the fourth and fifth decades. In men, these tumours are rare and inflammation is more common because of the hairy skin. Owing to the specific location of these tumours, it was necessary to perform proper diagnostic tests to confirm the assumed nature of the tumours and exclude possible communication with the spinal canal. Intraoperatively, lipomas may be seen as soft, yellow, shiny, smooth, mobile, encapsulated and, occasionally, lobulated subcutaneous masses. Microscopically, the lesions show lobular growth of mature adipocytes with demarcated borders, a fibrous capsule and a central vacuole.[5] Most lipomas pose no diagnostic dilemmas. However, when presented with large (>10 cm) or rapidly growing masses, especially of the head and neck region, one should be concerned about a malignancy.[9] The main diagnostic dilemma is to distinguish a lipoma from a liposarcoma. Rarely, lipomas can also become malignant or, from the beginning, they can be liposarcomas.[6] There is also chance of having complications like bleeding pressure ulcer with this kind of giant lipoma in the neck region.[7] Removal of those tumours is not difficult because of a clear demarcation of the surrounding tissues. Improved diagnostic imaging technology [such as computed tomography (CT) or magnetic resonance imaging (MRI)] has been accompanied by increasing reports of the utility of these imaging techniques in the diagnosis of complex or unusual neck masses. On CT scans, lipomas have the typical characteristics of homogeneous masses with few septations, a specific range of CT Hounsfield Unit (HU) values (usually between -50 and -150 HU), and they show no contrast enhancement.[9] MRI can also accurately diagnose lipomas pre-operatively by comparison of signal intensity on T1- and T2-weighted images.[8] Moreover, the margin of a lipoma is clearly defined by MRI as a “black-rim”, enabling lipomas to be distinguished from the surrounding adipose tissue, a distinction that cannot be made from CT images.[8]

Complete excision is the treatment of the choice for giant lipoma. Liposuction for such tumours has also been reported. Because giant lipomas usually have a well-defined pseudocapsule, dissection around these benign neoplasms is performed rather easily.[9]

In conclusion, giant lipoma in the neck region is rare and complications like bleeding decubiitus ulcer and malignant transformation are important complications. Before surgery, CT or MRI neck is an essential imaging study. Early surgical intervention is a must for these giant lipomas.
